# Arc Discharge System for Micromachining of Helical Fiber

**DOI:** 10.3390/mi14061120

**Published:** 2023-05-26

**Authors:** Jian Wang, Chao Ma, Shaochen Duan, Donghui Wang, Libo Yuan

**Affiliations:** 1 School of Optoelectronic Engineering, Guilin University of Electronic Technology, Guilin 541004, China; 2 Key Laboratory of In-Fiber Integrated Optics, Ministry of Education of China, Harbin Engineering University, Harbin 150001, China

**Keywords:** four-electrode method, electric arc, large constant-temperature field, helical fiber

## Abstract

This article developed a micromachining system of arcing helical fiber with four electrodes to address the issues with conventional approaches to processing helical fibers, which have several uses. The technique may be utilized to create several types of helical fibers. First, the simulation demonstrates that the four-electrode arc’s constant-temperature heating area is larger than the two-electrode arc’s size. A large constant-temperature heating area is not only beneficial to the stress release of fiber, but also reduces the influence of fiber vibration and reduces the difficulty of device debugging. Then, a variety of helical fibers with various pitches were processed using the system presented in this research. By using a microscope, it can be observed that the cladding and core edges of the helical fiber are constantly smooth and the central core is tiny and off-axis, both of which are favorable for the propagation of optical waveguides. A low off-axis has been shown to minimize optical loss through modeling of energy coupling in spiral multi-core optical fibers. The transmission spectrum findings indicated that the device’s insertion loss and transmission spectrum fluctuation were both minimal for four different types of multi-core spiral long-period fiber gratings with intermediate cores. These prove that the spiral fibers prepared by this system have excellent quality.

## 1. Introduction

Helical fiber is a distinctive and important periodic microstructure fiber [1]. Helical fibers with large pitch and offset values have been used in various sensors since early times [2]. Notable is the use of large-bias helical fibers, also known as crimped fibers, to illustrate topological phase effects in optical systems. The optical Berry phase was first discovered in this type of fiber [3]. Sensors, circular polarizers, couplers [4,5,6], optical vortex generators [7,8], and lasers [9] may all make use of spiral fibers. In [10,11], it was demonstrated that light, when transmitted in the fiber with a very large central core encircled by helical side cores, causes loss of higher-order core modes. Moreover, helical photonic crystal fibers have been developed, and their potential uses as filters, dispersion controllers [12], sensors [13], and spin-orbit couplers [14] have all been investigated.

In recent years, eccentric-core and multi-core helical fibers have been employed to develop helical-core surface plasma resonance (SPR) fiber sensors and to examine the spin-orbit coupling impact of the optical field transmitted down the helical waveguide [15,16]. A flexible side-throw helical-core fiber SPR sensor with a compact structure and controllability of the resonance wavelength and sensitivity was proposed in [17] based on the helical-core fiber created by eccentric-core fiber. The cladding whispering-gallery modes (WGM) explain how the radiation field of the helical-core fiber activates SPR. The findings demonstrate that WGM is highly responsive to changes in torsional pitch, allowing the resonant wavelength of the SPR sensor to be successfully altered and the sensor’s sensitivity to be managed. The experiment shows that the adjustment method can achieve higher sensitivity, especially for short-pitch adjustments. This type of SPR sensor is distinct from the standard SPR sensor based on curved fiber, which has a large bending loss. The optical field can propagate stably in the straight fiber cladding of the spiral-core fiber SPR sensor. Therefore, multiple sensor arrays with different resonant wavelengths and sensitivities can be obtained in the same helical-core fiber by manufacturing multiple different pitches [18]. These features make the sensor array a single-core or multi-core optical fiber with different pitches in producing for a multi-parameter measuring sensor array and may be widely used in chemical/biological sensors.

In 1979, Ulrich and Simon realized the circular birefringence of light in single-mode fibers by using the mechanical torsion method, and they also applied chirality to fiber for the first time [19]. Numerous spiral-fiber preparation techniques have emerged in recent years. There are two methods to prepare spiral fiber. The first method is to rotate the prefabricated rod at a high speed in the process of fiber drawing. The second method is to heat and twist the fiber twice based on the drawn fiber. The commonly used heating methods of the second preparation method are hydrogen and oxygen flame [20], CO_2_ laser [21], and arc discharge [22]. Hydrogen and oxygen flame has the advantage of a wide heating area and uniform heating temperature, but it has the drawback of requiring an electrolytic electrolyte to produce the hydrogen and oxygen needed for high-temperature heating, and hydrogen itself is quite toxic. The CO_2_ laser has flexible and prepared high-quality helical fiber benefits, but its disadvantages are being expensive and having strict requirements on the optical path. Although the temperature zone is very small, the classic arc discharge has the advantages of flexibility, simplicity, and low cost.

Considering the potential applications of spiral fiber and the shortcomings of traditional two-electrode systems, a micromachining system of arc spiral fiber with four electrodes is studied in this paper. First, a simulation is used to compare the four-electrode arc’s temperature field to the conventional two-electrode arc. The following research and discussions were conducted to evaluate the performance of the system developed in this article: To comprehend the structure of the spiral fiber prepared by the system developed in this article, a variety of spiral fiber devices were prepared using the system and observed under a microscope. The benefits of the system’s low off-axis spiral fibers were described by going through the coupling condition of multi-core spiral devices. Four different types of multi-core spiral long-period fiber gratings (HLPFGs) with intermediate cores were made, and their transmission spectra were examined to access the effectiveness of the system.

## 2. Principle and Simulation

### 2.1. Working Principle and Process of the Four-Electrode Arc’s Helical Fiber Micromachining System

**Figure 1:**
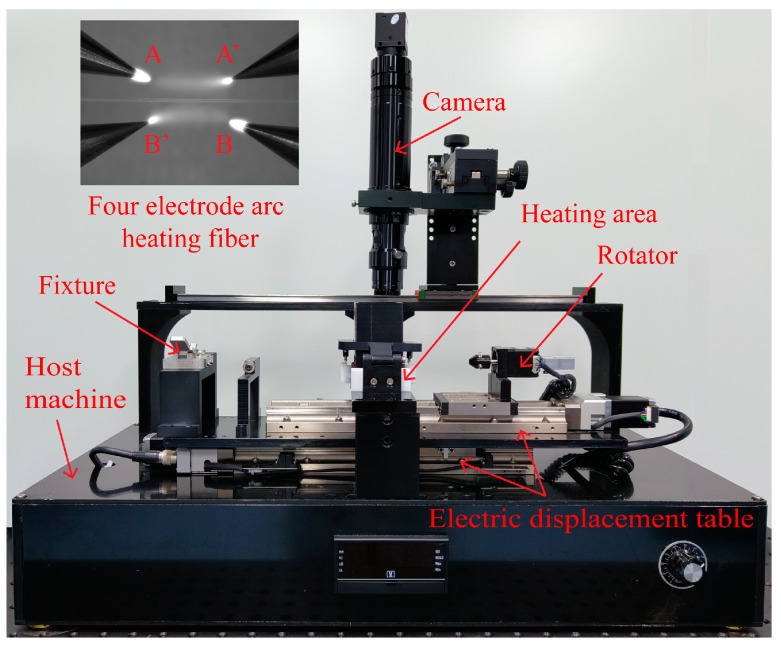
Structure of four-electrode arc helical fiber micromachining system.

The innovative arc helical fiber micromachining system is depicted in Figure 1 as having a four-electrode arc heating area, camera, electric displacement table, electric rotary table, fixing fixture, and host machine. The four-electrode arc heating area is mainly used for optical fiber heating. The essential components of the region, in addition to the four-electrode heating module, are the windshield and fiber optic elastic pin. The windshield is used to prevent the influence of airflow on the arc. To lessen the vibration of the processed fiber during processing, the fiber elastic presser is pressed into the processed fiber’s V-shaped groove. The camera can assist in changing the location of the four electrodes in addition to being utilized to view the machining of the helical fiber. The electric rotating table is used to rotate the fiber in the processing process. The module includes a rotating electrode, fiber clamp, and ferrule insert to prevent the swing of the fiber. The motorized rotary table’s stopper screw is utilized to precisely adjust the ferrule’s location. By fixing one end of the fiber, the fixing fixture makes the processed optical fiber properly taut. The electric displacement platform is used to move fiber during fiber processing. The host computer’s duties include creating an arc at the four electrodes, controlling the arc’s size, and managing the operation of other system components.

**Figure 2:**
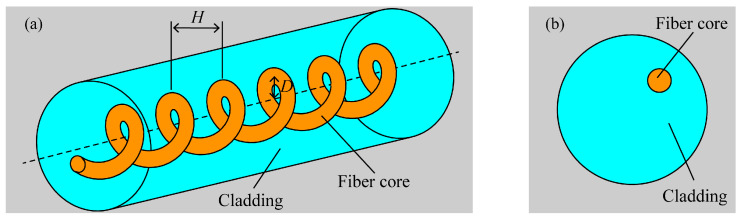
The structure of the helical-core fiber: (**a**) geometric structure diagram of helical-core fiber with pitch *H* and core offset *D*; (**b**) cross-sectional diagram of the helical-core fiber.

The following steps are taken to prepare spiral optical fibers: initially, the coating layer is removed from the optical fiber’s processed processing component. The optical fiber has one end fastened with a fixed fixture and the other end fixed with a fixture to the electric rotary table. An elastic presser foot is used to press the portion of the optical fiber that has been stripped of its coating layer into the V-shaped groove within the four-electrode arc heating zone. The fiber is straightened during processing by suspending a little counterweight from one end close to the fixed fixture. After the preliminary work is finished, the host program can be used to set the rotation speed of the electric rotary table, the displacement speed of the electric displacement table, and the temperature field of the four-electrode arc discharge to process spiral optical fibers. The fiber’s spiral structure is shown in Figure 2. Spiral fiber optic devices are often created by modulating the fiber core axially in a spiral pattern, where *H* represents pitch and *D* represents off-axis amount.

### 2.2. High-Temperature Heating Field of the Four-Electrode Arc

The system’s four electrodes’ axes and the fiber’s axes are both situated in the same plane. Figure 1 depicts the configuration of electrode A, electrode A’, electrode B’, and electrode B. A set of arc discharge paths is formed by electrodes A and A’, while another set is created by electrodes B and B’. There are around 3 mm between electrode A and electrode A’, roughly 1.6 mm between electrode A and electrode B, and the electrode axis and fiber axis are acutely angled at 20°. The two groups of electrodes are powered by a 50 Hz high-voltage AC power supply, and the two groups of electrodes are switched on alternately at the positive half cycle and the negative half cycle of the AC power supply. The discharge current flows alternately through electrode A to electrode A’ and through electrode B to electrode B’. The two groups of electrodes are, respectively, switched on to form a large constant-temperature fiber heating area [23].

**Figure 3:**
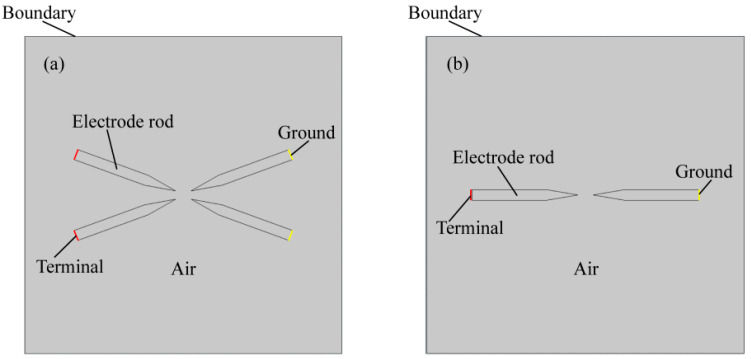
(**a**) Geometric model for temperature field simulation of four-electrode arc; (**b**) geometric model for temperature field simulation of two-electrode arc.

Laminar flow, fluid heat transfer, current flow, and magnetic field are the four physical fields that are coupled in the intricate process known as arc. They may be coupled to create an arc simulation model made up of mass-conservation, momentum-conservation, and energy-conservation equations [24]. A two-dimensional simulation model of the arc is created using the finite element approach to compare the temperature fields created by the four and two electrodes, as illustrated in Figure 3. The four-electrode simulation architecture is as shown above in the simulation geometry model, which includes electrodes, air, simulation border, total end, and ground. Two parallel electrodes are simulated at a distance of 3 mm from one another.

The simulation conditions were set as follows:

(1) Laminar flow conditions:

The surface of the electrodes was set to a non-slip boundary condition, and the air acceptor force was affected by the Lorenz force. The initial pressure was 101.325 kPa and the initial velocity field was 0 m/s.

(2) Fluid heat transfer conditions:

The electrodes were solid, the air was fluid, the initial temperature was 293.15 K, the electrodes were heat insulated, and the simulated boundary was open.

(3) Current conditions:

The terminal was connected to the circuit and AC was applied to it. All areas of the simulation geometry model followed the current conservation; the initial potential was 0 V, and the simulation boundary as set to electrical insulation.

(4) Magnetic field conditions:

The initial vector magnetic potential was 0 Wb/m, and the simulation boundary was set to magnetic insulation.

**Figure 4:**
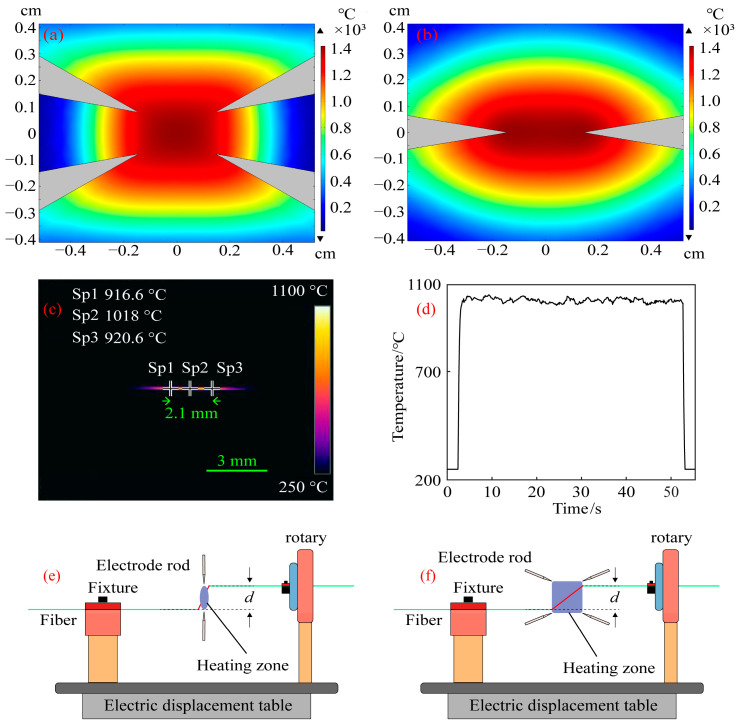
(**a**) Four-electrode temperature zone simulation diagram; (**b**) Two-electrode temperature zone simulation diagram; (**c**) Temperature maps taken by infrared cameras; (**d**) The highest temperature on the fiber being processed; (**e**) Schematic diagram of two-electrode arc temperature zone processing fiber. (**f**) Schematic diagram of four-electrode arc temperature zone processing fiber.

The four-electrode simulation was set up in the manner described above, and the simulation distance for the two-electrode electrode was 3 mm to compare the temperature field created by the four-electrode arc with that created by the two-electrode arc. Through the finite element method, Figure 4a,b were obtained. Taking the highest temperature point on the processed fiber as the reference point, two temperature points with a change of 100 °C relative to the reference point were found on the heated fiber, and the distance between the two temperature points was defined as the length of the constant-temperature zone of the heated fiber. The heating zone of the four electrodes is square and the length of constant-temperature heating is 3.08 mm, while the heating zone of the two electrodes is oval and the length of constant-temperature heating is 1.98 mm, as shown in Figure 4a,b. By calculation, the constant-temperature heating zone of four electrodes is about 1.56 times that of two electrodes. In addition to helping the heated fiber release tension and soften, a large, consistent-temperature heating region also lessens the impact of fiber vibration and the challenge of device debugging. To understand the temperature during the actual processing of the spiral fiber, an infrared camera was used to photograph the temperature during the processing of fiber with four electrodes, as shown in Figure 4c,d, respectively. When the maximum temperature of arc formation with four electrodes fluctuates around 1030 °C, the length of the constant-temperature zone is about 2.1 mm.

There is a slight height difference at the two ends of the fiber heating temperature zone due to the equipment’s small processing errors and movement during installation and preparation, which causes spiral modulation between the fiber cladding and the intermediate core when the fiber is twisted. The traditional two-electrode system has a narrow heating temperature zone, as shown in Figure 4e. The off-axis of the helical structure of the optical fiber center core and the cladding may be described as *d* [22] for the fiber with an intermediate core when the height difference between the two ends of the heating zone is *d*. Second, the treated fibers must be moved during the spiral optical device processing, which unavoidably results in a minor vibration from the processed fibers. The processed helical optical fiber will not be heated evenly enough and will not be smooth enough if the thermostatic zone of the optical fiber heating is not big enough. As can be seen in Figure 4f, the helical optical fiber processing system with a large constant-temperature zone described in this research is more suited to stress release and optical fiber softening. Therefore, the off-axis amount of the produced helical optical device will be smaller than *d* when the height difference is also *d*. Additionally, the system can smooth out spiral optical fiber devices by reducing the influence of optical fiber vibration during processing thanks to the broad constant-temperature zone. The smooth surface of the helical optical fibers produced using hydrogen-oxygen flame processing with a large thermostatic zone in [25] also quite supports the claim that a larger thermostatic heating region can provide a smoother surface for helical optical fibers.

### 2.3. Modulation of the Refractive Index of the Multi-Core Fiber Core

The modulation of the refractive index of the multi-core fiber core is divided into two parts: the change of the refractive index of the multi-core fiber core and the spiral modulation of the fiber core along the axis.

**Figure 5:**
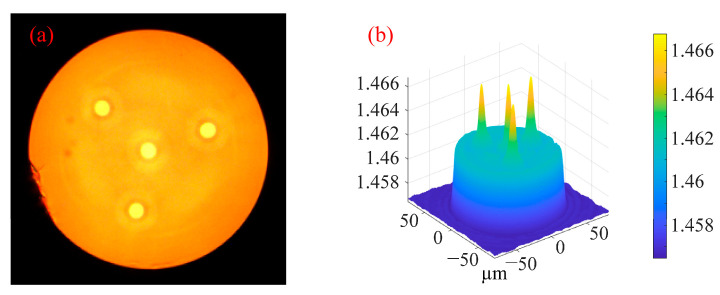
(**a**) Cross section of triangular four-core fiber; (**b**) 3D refractive index distribution of triangular four-core fiber at 532 nm wavelength.

**Figure 6:**
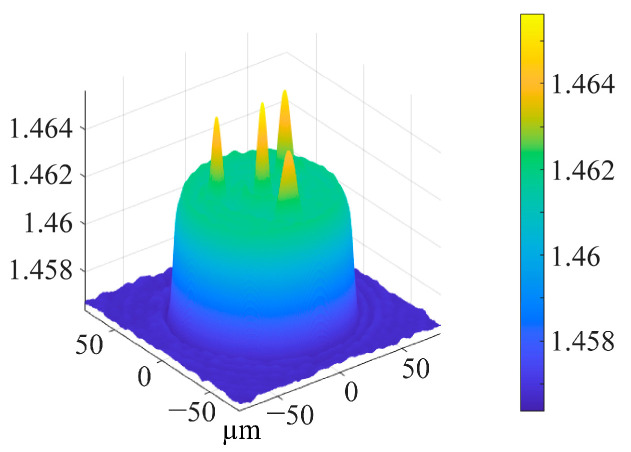
Three-dimensional refractive index distribution of triangular four-core fiber at 532 nm wavelength after processing.

Multi-core spiral fiber devices’ processing temperatures correspond to the fiber’s softening temperature. Too little warmth prevents the produced fiber from twisting into a spiral configuration. When the temperature is too high, the treated fiber melts because it has reached its melting point. At this moment, gravity and other elements can readily impact the fiber during spiral processing, which is also unfavorable to the processing of the fiber’s spiral structure. Using triangular four-core fibers as an example, pertinent experiments were carried out in order to investigate the changes in the refractive index of the core following spiral processing of multi-core optical fibers. Figure 5 displays the cross-section and refractive index distribution of triangular four-core optical fibers. The temperature depicted in Figure 4c,d corresponds to the softening temperature of the triangular four-core fiber during spiral processing. After processing, Figure 6 shows the triangular four-core fiber’s three-dimensional refractive index profile. The triangular four cores’ refractive index has changed just a little, by around 0.001, as can be observed by contrasting Figure 5 and Figure 6. Possible explanations for tiny adjustments include: slight thermal diffusion caused by heating; a small adjustment in the optical fiber’s refractive index owing to the spiral configuration. Each fiber core produces a spiral structure brought on by the twisting of the fibers and the height difference between the two ends of the constant-temperature zone in addition to variations in refractive index. As a result, this technology is capable of producing spatial modulation of each fiber core’s refractive index in multi-core fibers.

## 3. Results and Discussion

### 3.1. Microscopic Images of Prepared Helical Multi-Core Fibers

**Figure 7:**
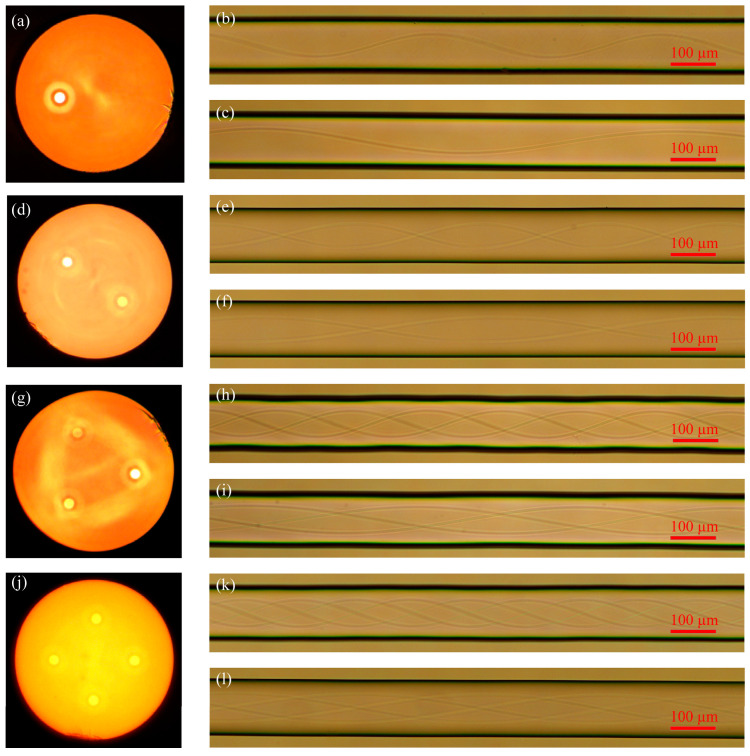
Cross-sectional diagram of multi-core fibers and lateral diagram of the helical multi-core fibers without a central core: (**a**) Cross-sectional diagram of the eccentric-core fiber; (**b**) Eccentric-core helical fiber with 500 μm pitch; (**c**) Eccentric-core helical fiber with 1000 μm pitch; (**d**) Cross sections of symmetrical dual-core fiber; (**e**) Symmetrical double-core helical fiber with 500 μm pitch; (**f**) Symmetrical double-core helical fiber with 1000 μm pitch; (**g**) Cross section of triangular three-core fiber; (**h**) Triangular three-core helical fiber with 500 μm pitch; (**i**) Triangular three-core helical fiber with 1000 μm pitch; (**j**) Cross section of square four-core fiber; (**k**) Square four-core helical fiber with 500 μm pitch; (**l**) Quad-core helical fiber with 1000 μm pitch.

As can be seen from Figure 7, this paper adopts fiber without a central core and uses the developed system to prepare spiral fiber with different pitches. The cladding edge of helical fiber without a central core is smooth, and the structure change of the cladding without a central core fiber during spiral processing cannot even be seen when looking at the cladding edge of spiral fiber without a central core via a microscope. It is shown that the system has little effect on the fiber cladding without a central core in the process of preparing helical fiber. The small change of the cladding of helical fiber without a central core has little effect on its optical waveguide. By observing the core structure of spiral fiber without a central core with a microscope, the torsional structure of the core of different helical fibers is clear, visible, continuous, and smooth. The above characteristics are conducive to the propagation of light, such as reducing the generation of unnecessary modes and reducing the loss of light.

**Figure 8:**
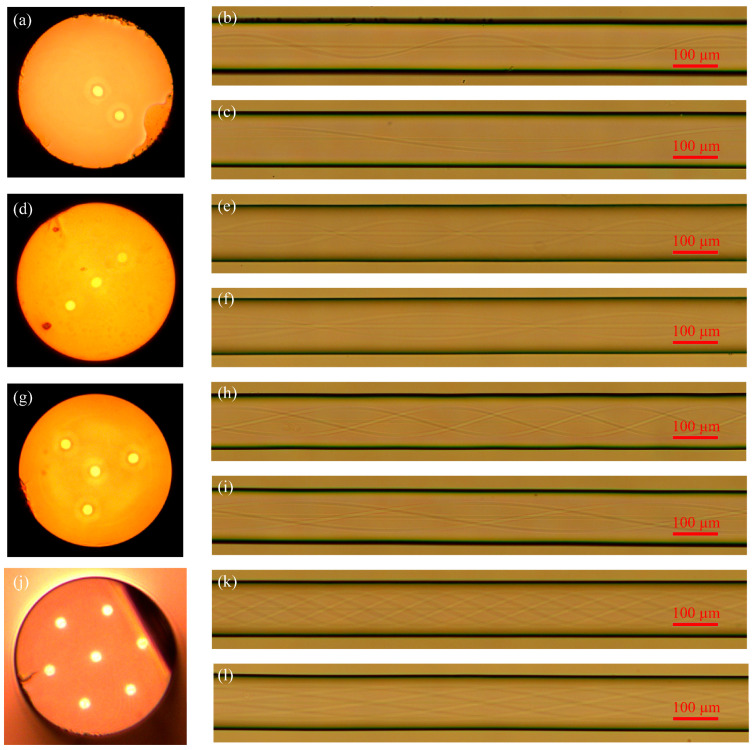
Cross-sectional diagram of multi-core fibers and lateral diagram of the helical multi-core fibers with a central core: (**a**) Cross-section diagram of biased-dual-core fiber; (**b**) Biased-dual-core fiber with 500 μm pitch; (**c**) Biased-dual-core fiber with 1000 μm pitch; (**d**) Cross sections of linear three-core fiber; (**e**) Linear three-core helical fiber with 500 μm pitch; (**f**) Linear three-core helical fiber with 1000 μm pitch; (**g**) Cross sections of triangular four-core fiber; (**h**) Triangular four-core helical fiber with 500 μm pitch; (**i**) Triangular four-core helical fiber with 1000 μm pitch; (**j**) Cross sections of seven-core fiber; (**k**) Seven-core helical fiber with 500 μm pitch; (**l**) Seven-core spiral fiber with 1000 μm pitch.

Figure 8 shows how the developed technology was applied in this work to produce multi-core helical fibers with a center core of different pitches. Using a microscope to examine the helical fibers’ core structures, the torsional structure of the fiber core can be seen. The continuously smooth structure of the torsional non-intermediate core is the same as that of helical fiber without a central core. The twisting center of the core has a tiny off-axis distance in addition to a smooth and continuous construction and no obvious machining traces. The aforementioned properties of multi-core spiral fibers with intermediate cores, such as their low overall loss of light when transmitted in the intermediate core of spiral fiber devices, are advantageous for the transmission of necessary light. This technique produces spiral fibers of various pitches and kinds with equally outstanding outcomes, demonstrating its stability.

### 3.2. Optical Coupling between Multi-Core Fiber Cores

**Figure 9:**
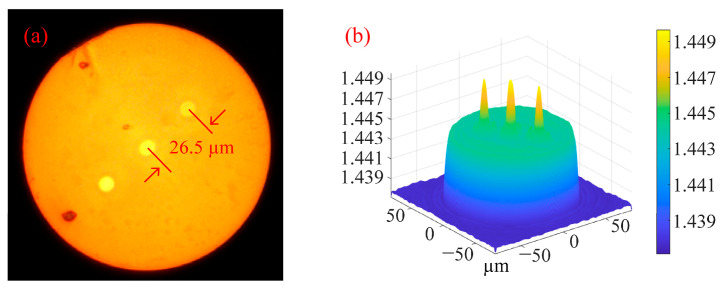
(**a**) Cross section of straight three-core fiber; (**b**) 3D refractive index distribution of straight three-core fiber at 1550 nm wavelength.

The distance between the cores of multi-core optical fibers is typically rather large, and sometimes an isolation layer is placed between the cores to avoid coupling between the cores. To better understand the coupling situation between the cores of multi-core spiral fibers and understand the performance of the system studied in the paper, a straight three-core fiber is used as an example to simulate the coupling situation using the beam propagation method. The fiber cross-section and 3D refractive index dispersion are displayed in Figure 9.

**Figure 10:**
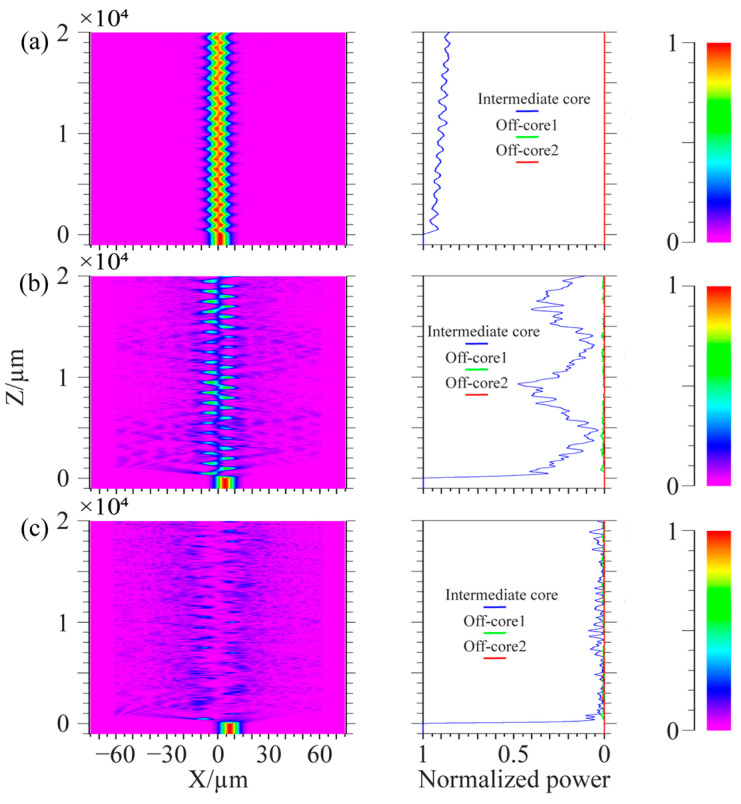
Coupling process of linear spiral three-core fiber with a cycle of 1000 μm. (**a**) Off-axis quantity is 1 μm; (**b**) Off-axis quantity is 4 μm; (**c**) Off-axis quantity is 7 μm.

**Figure 11:**
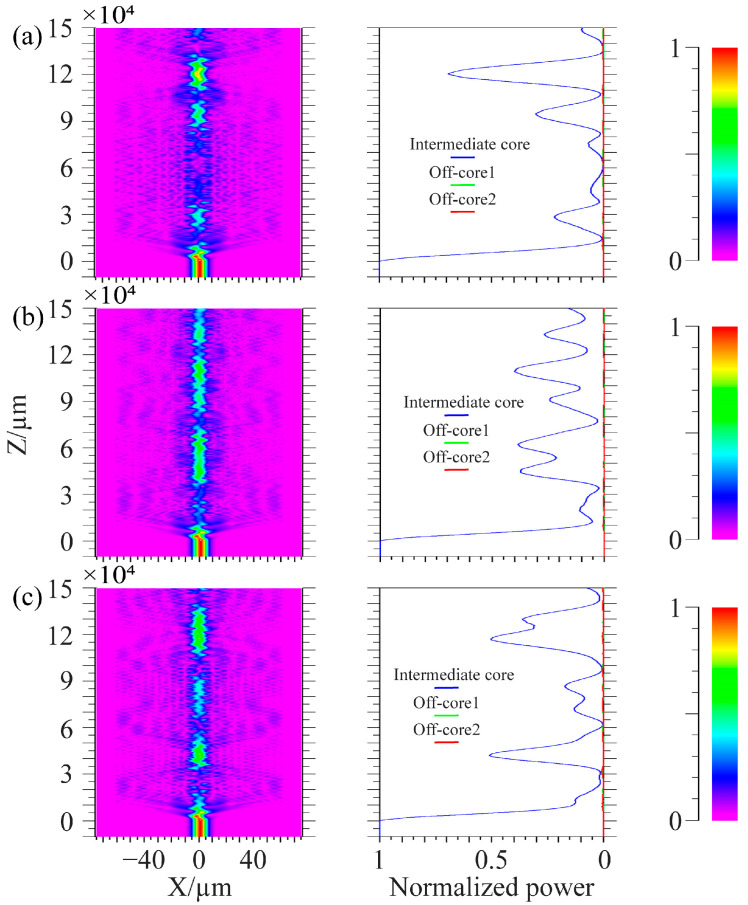
Coupling process of linear spiral three-core fiber with the off-axis quantity of 1 μm. (**a**) The cycle is 536 μm; (**b**) The cycle is 500 μm; (**c**) The cycle is 469 μm.

In the simulation, the center core of the spiral fiber is connected to a 1000 μm single-mode fiber connection, where light is input from the single-mode fiber, to better inject light into the spiral fiber. The wavelength of injected light is 1.55 μm. The simulation parameters in Figure 10 are: the core refractive index is 1.449, the cladding refractive index is 1.444, the core diameter is 8.7 μm, the diameter of the cladding is 125 μm, the cycle is 1000 μm, and the off-axis values of the intermediate core in Figure 10a–c are 1 μm, 4 μm, and 7 μm. The simulation parameters in Figure 11 are: the core refractive index is 1.449, the cladding refractive index is 1.444, and the core diameter is 8.7 μm. The diameter of the cladding is 125 μm; the cycles in Figure 11a–c are 536 μm, 500 μm, and 469 μm; and the off-axis amount of the middle core is 1 μm. Spiral linear three-core optical fibers should each have their energy monitored independently.

Figure 10 shows that the light in the edge core and center core does not couple at tiny off-axis distances. Only the core mode of the intermediate core and the cladding mode of the optical fiber are coupled; when the off-axis quantity rises, the middle core’s optical energy loss rises and may even entirely radiate out. The light in the edge core and the center core only weakly couple together because of the long distance between the fiber cores. It is clear from the manuscript’s third section’s conclusion that the cladding and intermediate core processed by the system under study in this work have a negligible amount of off-axis. The technology examined in this work may successfully decrease the optical loss brought on by excessive off-axis when utilized in conjunction with the simulation findings in Figure 10. From the conclusion in the third part of the paper, it can be seen that the cladding and intermediate core of the fiber processed by the system have relatively small off-axis values. From Figure 11, it can be seen that when the period of preparing spiral fiber devices in the system is perturbed, it only affects the coupling length of the light and has little effect on the overall loss caused by optical coupling. The energy coupling between fiber cores remains small at small cycles. Comparing Figure 10a with Figure 11, when the off-axis amount is the same, the core energy loss of spiral fiber devices with smaller cycles is greater.

### 3.3. Multi-Core HLPFGs with an Intermediate Core

In this study, the manufactured spiral fiber is processed into a spiral long-period fiber grating, and its quality is assessed using transmission spectroscopy. This is due to the fact that spiral long-period fiber gratings may also be used to assess the off-axis magnitude and continuous smoothness of spiral fiber devices. It may also be utilized to assess the processing stability of the system examined in the study because of the periodic spiral structure. Figure 5 shows spiral multi-core fibers without an intermediate core. Due to the large distance between the edge core and the center of the optical fiber, the off-axis of the edge core structure is relatively large in order to exclude the influence of large off axis quantities on spectral results, so that spiral multi-core fibers without intermediate cores are not inappropriate for processing long-period gratings to verify device quality. Because of its inherent structure, the intermediate core of a multi-core fiber with an intermediate core will not significantly change shape during processing. Therefore, in order to better evaluate the quality of the processed spiral fiber, this paper prepared the intermediate core of the multi-core fiber into a long-period grating to evaluate the quality of the spiral fiber device.

**Figure 12:**
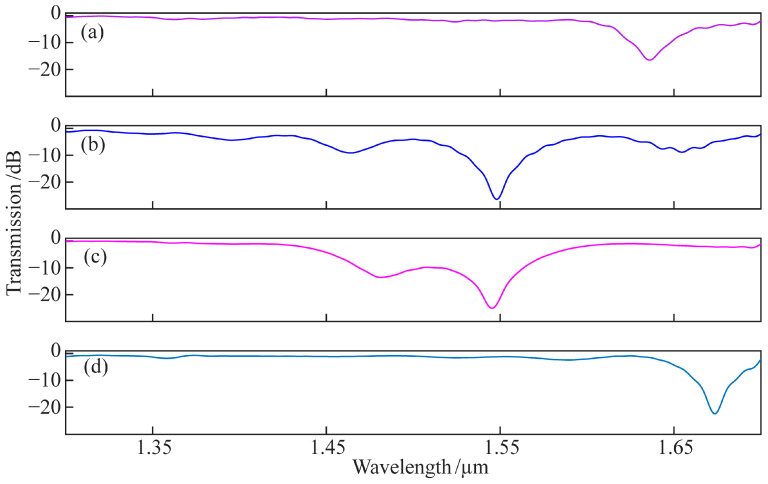
The spectra of the multi-core HLPFGs: (**a**) HLPFG of biased-dual-core fiber with 1100 μm pitch; (**b**) HLPFG of linear three-core fiber with 800 μm pitch; (**c**) HLPFG of cross sections of triangular four-core fiber with 750 μm pitch; (**d**) HLPFG of seven-core fiber with 750 μm pitch.

Four kinds of multi-core HLPFGs with an intermediate core were prepared. Figure 12 shows the spectra of four kinds of multi-core HLPFGs. The average loss at wavelengths between 1.2 μm and 1.35 μm is currently employed as the insertion loss of the gratings to fully describe the insertion loss of multi-core HLPFGs. The four gratings’ transmission spectra correspond to insertion losses of 0.411 dB, 1.3786 dB, 0.4394 dB, and 0.122 dB, respectively. The magnitude of grating fluctuations is calculated as the difference between the highest and minimum values of the transmission spectrum intensity at wavelengths between 1.2 μm and 1.35 μm. The transmission spectra of the four gratings correspond to maximum fluctuations of 1.7264 dB, 1.363 dB, 1.104 dB, and 1.4054 dB, respectively.

In order to illustrate the performance of the four-electrode arc spiral fiber micromachining system in this paper, the results of the relevant literature and this work are presented in Table 1. The four-electrode arc discharge method for producing multi-core HLPFGs with an intermediate core described in this paper not only achieves a minimum spectral loss of less than 1 dB, but it is also easy to use, flexible, inexpensive, and has a wide constant-temperature range. This work offers a practical technique for making high-grade multi-core spiral fiber devices.

## 4. Conclusions

The four-electrode arc micromachining system has a broader constant-temperature zone than the conventional two-electrode arc system. The huge constant-temperature heating zone not only helps the heated fiber relax and soften, but it also lessens the impact of vibration on the fiber and makes device debugging easier. Observation and experimentation reveal that the helical fiber produced by the technique described in this study is of good quality and may be utilized to create a variety of multi-core helical fibers, indicating a wide range of potential applications. The single-eccentric-core SPR sensor, the multi-core helical fiber interferometer, the multi-core helical long-period fiber grating, and the multi-core helical fiber coupled with a Bragg grating deformation sensor are a few examples.

## Figures and Tables

**Table 1:** Comparison of different preparation methods for the same device structure.

Device Structure	Preparation Method	Minimum Loss of Transmission Spectrum (dB)	Advantages and Disadvantages	Reference
Intermediate-core spiral long-period fiber grating	CO_2_ laser	>1	Advantages: Flexible and high-quality. Disadvantages: Cumbersome debugging of the optical path and expensive.	[26]
		>1		[27]
		>1		[28]
	Oxyhydrogen flame	≈1	Advantages: Wide heating area and uniform heating temperature.	[29]
		>1	Disadvantages: Danger of using hydrogen.	[30]
	Two-electrode arc discharge	>1	Advantages: Simple and flexible.	[22]
		>1	Disadvantages: Narrow constant-temperature zone.	[31]
	Four-electrode arc discharge	<1	Advantages: simple and flexible, large constant-temperature zone, and cheap price.	This work
			Disadvantages: The arc needs further optimization	

## Data Availability

Not applicable.
